# New Insights into a Vanadium Precipitation Process by Hydrolysis Based on Manganese Salt Pretreatment

**DOI:** 10.3390/ma17246223

**Published:** 2024-12-20

**Authors:** Mengxia Liu, Tao Jiang, Jing Wen, Zibi Fu, Tangxia Yu, Guangdong Yang, Sanyuan Xia, Hao Xiao

**Affiliations:** 1 State Key Laboratory of Vanadium and Titanium Resources Comprehensive Utilization, Panzhihua 617000, China; 13982377105@163.com; 2 School of Metallurgy, Northeastern University, Shenyang 110819, China; 15265373295@163.com (M.L.); wenjing@smm.neu.edu.cn (J.W.); 2210665@stu.neu.edu.cn (T.Y.); 2110566@stu.neu.edu.cn (G.Y.); 13739801061@163.com (S.X.); 15101851894@163.com (H.X.)

**Keywords:** sodium metavanadate solution, manganese vanadate, acid dissolution, vanadium precipitation by hydrolysis

## Abstract

Vanadium precipitation is the key step in producing vanadium products from vanadium solution. The sustainable development of the vanadium industry requires new environmentally friendly processes for vanadium precipitation. In this study, NaVO_3_ solution was pretreated with manganese salt to preliminarily separate the vanadium and sodium components. The product of vanadium extraction by manganese salt was dissolved by acid to produce manganese vanadate solution. After vanadium precipitation by hydrolysis, manganese removal, and calcination, the target product V_2_O_5_ was obtained. Scanning electron microscopy (SEM), X-ray diffraction (XRD), inductively coupled plasma emission spectrometry (ICP-OES), and atomic absorption spectrometry (AAS) were used to perform the characterization and analyses. The results showed that vanadium and manganese have a strong binding ability. The rate of vanadium extraction by manganese salt reached 99.75%, and the product of vanadium extraction by manganese salt was Mn_2_V_2_O_7_, with a sodium content of only 0.089%, confirming the effective separation of vanadium and sodium. The acid dissolution rate of the vanadium extraction product reached 99.95%, and the rate of vanadium precipitation by hydrolysis reached 97.87%. After manganese removal and calcination, the purity of the V_2_O_5_ product reached 98.92%. In addition, the recyclability of manganese sulfate and ammonium sulfate was analyzed. The process reduced the production of ammonia–nitrogen wastewater, laying a foundation for researching new technologies for extracting vanadium from vanadium slag.

## 1. Introduction

Vanadium is an indispensable nonferrous metal, an important strategic resource in modern national defense and industry [1,2]. Vanadium slag is obtained by smelting vanadium–titanium magnetite in a blast furnace and blowing vanadium in a converter. V_2_O_5_ is prepared by extracting vanadium from vanadium slag, which is widely used in steel, the chemical industry, medical treatments, aerospace, new energy, and other fields [3,4,5,6,7,8,9,10]. This wide usage necessitates establishing an industrial chain for the clean and efficient preparation of V_2_O_5_ products for the utilization of vanadium resources and the development of the vanadium industry.

The roasting and leaching process group of vanadium slag includes sodium roasting–water leaching, calcification roasting, and manganese roasting–acid leaching [11,12,13]. The sodium roasting–water leaching process is widely used because of its high leaching rate. NaVO_3_ [14,15,16] is the main vanadium-containing phase in the sodium roasting clinker of vanadium slag, with its leaching solution obtained through water immersion. Vanadium pentoxide is mainly prepared from a vanadium-containing leaching solution through chemical precipitation, ion exchange, and extraction [17,18,19,20,21]. However, the ion exchange and extraction methods are relatively complex and costly, making them more suitable for experimental studies on vanadium extraction rather than large-scale industrial applications. Hence, the chemical precipitation method is widely used because of its short process, simple operation, and low production cost.

The chemical precipitation method mainly includes the ammonium salt precipitation and hydrolysis precipitation methods [22,23,24,25,26,27,28]. Xiong [29] studied a vanadium-containing solution with a vanadium concentration of about 18 g/L and a sodium concentration of about 23 g/L. Vanadium was precipitated by ammonium salt at a pH of 8.0, and the precipitation rate was 99.52%. The sodium impurity in the V_2_O_5_ product was high, leading to a purity of only 92.37%. At a pH of 2.0, reaction temperature of 90 °C, and reaction time of 60 min, the vanadium precipitation rate and product purity could exceed 99%. However, the ammonium salt precipitation method cannot separate ammonium and sodium, leading to the generation of a considerable amount of refractory ammonia–nitrogen wastewater, which pollutes the environment and affects the ecological balance [30,31,32,33]. The hydrolysis precipitation method is more environmentally friendly than the ammonium salt precipitation method, but it has a low vanadium precipitation rate and produces low-purity vanadium products, which is due to the sodium ion concentration in solution. Wu [34] studied the hydrolysis and precipitation of the leaching solution of sodium roasting of vanadium–chromium slag. The vanadium concentration of the solution was 17.86 g/L, and the sodium concentration was 54.80 g/L. At a reaction pH of 1.8, reaction temperature of 95 °C, and reaction time of 60 min, the vanadium precipitation rate reached 91.61%, and the sodium content in the vanadium products was approximately 7.71%. Meanwhile, the process of sodium ion concentration in the solution from 35.2 to 90 g/L was studied, and the vanadium precipitation rate showed an obvious downward trend. Evidently, an excessively high sodium concentration adversely affects both the vanadium precipitation rate and the product purity in the processes of alkaline ammonium salt precipitation and vanadium precipitation by hydrolysis.

Therefore, if an additive is added to the sodium vanadate solution to separate vanadium and sodium, it would be crucial to improve the vanadium precipitation rate and enhance the product purity during the vanadium precipitation by hydrolysis. Wen [35] found that manganese has a strong binding capacity with vanadium. Under specific roasting conditions, vanadium and manganese combine to form Mn_2_V_2_O_7_, which dissolves in a sulfuric acid medium to yield a vanadium-containing solution, transforming vanadium from solid to liquid. This process establishes a foundation for the clean and efficient preparation of V_2_O_5_ products through the hydrolysis and precipitation of vanadium.

In this study, we used NaVO_3_ solution instead of a leaching solution of sodium-roasted vanadium slag to eliminate the influence of other impurity ions. We studied the effects of vanadium extraction conditions on the vanadium extraction rate of manganese salt, acid dissolution conditions on vanadium dissolution rate, and vanadium precipitation conditions on vanadium precipitation rate. The vanadium precipitation product was washed to remove manganese and calcined to obtain V_2_O_5_, and the purity of the related products was analyzed.

## 2. Experiment

### 2.1. Experimental Materials

NaVO_3_ (Macklin Bio-Chemical Co., Ltd., Shanghai, China, analytical purity > 99%) was used as the experimental raw material. MnSO_4_ (Fucheng (Tianjin) Chemical Reagents Co., Ltd., Tianjin, China, analytical purity > 99%) was used for vanadium extraction by manganese salt. H_2_SO_4_ (Sinopharm Chemical Reagent Co., Ltd., Beijing, China, analytical purity > 98%) was the acid dissolution leaching medium and was also used to adjust the pH during vanadium precipitation by hydrolysis. (NH_4_)_2_SO_4_ (Sinopharm Chemical Reagent Co., Ltd., Beijing, China, analytical purity > 99%) was used to wash and remove impurities in the product.

Other chemical reagents were used to titrate vanadium with standard ammonium sulfate. H_3_PO_4_ (Sinopharm Chemical Reagent Co., Ltd., Beijing, China, analytical purity > 85%) solution (50% volume fraction) was used to acidify the vanadium solution. KMnO_4_ (Sinopharm Chemical Reagent Co., Ltd., analytical purity > 99%) solution (20 g/L) was used to oxidize vanadium ions. NaNO_2_ (Sinopharm Chemical Reagent Co., Ltd., analytical purity > 99%) solution (20 g/L) and urea (Sinopharm Chemical Reagent Co., Ltd., analytical purity > 99%) solution (100 g/L) were used to reduce excess potassium permanganate. N-phenylanthranilic acid (Sinopharm Chemical Reagent Co., Ltd., analytical purity > 99%) solution (2 g/L) was used as a vanadium indicator. (NH4)_2_Fe(SO_4_)_2_ (Sinopharm Chemical Reagent Co., Ltd., analytical purity > 99.5%) solution (50 g/L) was used to reduce vanadium and other high-valent ions, and (NH_4_)_2_Fe(SO_4_)_2_ standard titration solution (0.005 mol/L) was used to titrate vanadium until the titration ended.

### 2.2. Experimental Process

We prepared a NaVO_3_ solution with a specific concentration and pH that are consistent with those of an actual vanadium slag sodium roasting leaching solution. NaOH solution was used to adjust the pH of the solution to 11 during the preparation.

#### 2.2.1. Vanadium Extraction by Manganese Salt

A 30 mL aliquot of the NaVO_3_ solution was placed in a conical flask, and a certain amount of MnSO_4_ was added. According to the experimental conditions outlined in Table 1, the content was placed in a constant-temperature water bath for the reaction to occur. After the reaction was completed, the resultant mixture was vacuum-filtered. The solid obtained from this filtration step was designated as the vanadium extraction product by manganese salt, which was used to perform characterization analysis and the subsequent acid dissolution process. A specified volume of the filtrate was designated as the waste liquor from the vanadium extraction by manganese salt; this volume was used to calculate the rate.

#### 2.2.2. Acid Dissolution

We placed 2 g of the vanadium extraction product by manganese salt into a three-necked flask and added 20 mL of deionized water. In accordance with the reaction conditions outlined in Table 1, the three-necked flask was positioned within a constant-temperature water bath pot, and H_2_SO_4_ was gradually added in a dropwise manner to adjust the pH of the solution. Upon completion of the reaction, vacuum filtration was performed on the mixture to obtain a specific volume of liquid, which was designated as the manganese vanadate solution and subsequently used in the vanadium precipitation by hydrolysis. Furthermore, the insoluble solid was dried to a constant mass at 110 °C for 24 h, and the acid solubility of the product was calculated by determining the difference in solid mass before and after the reaction.

#### 2.2.3. Vanadium Precipitation by Hydrolysis

We placed 40 mL of manganese vanadate solution, with a concentration of 17.29 g/L, into a three-necked flask. In accordance with the reaction conditions detailed in Table 1, the flask was positioned within a constant-temperature water bath to facilitate the reaction, and H_2_SO_4_ was gradually added to adjust the pH of the solution. Upon completion of the reaction, vacuum filtration was performed. The solid obtained was designated as the vanadium precipitation product by hydrolysis, which was subjected to characterization and calcination processes to yield the target product. The liquid obtained was designated as the waste liquor from vanadium precipitation by hydrolysis and used for calculating the rate of vanadium precipitation by hydrolysis.

**Figure 1:**
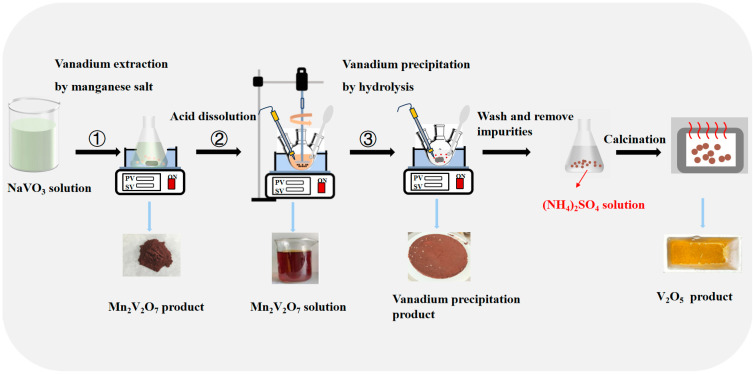
Experimental flowchart. (① Vanadium extraction by manganese salt; ② Acid dissolution; ③ Vanadium precipitation by hydrolysis).

The hydrolyzed vanadium precipitate was washed with (NH_4_)_2_SO_4_ solution in a conical flask [36]. The product was calcined in a muffle furnace at 550 °C for 2 h to obtain the V_2_O_5_ product. The experimental flow chart of this study is shown in Figure 1. The vanadium content in the solution is calculated using Equation (1), while the vanadium precipitation or dissolution rate is calculated using Equation (2).

(1) $$m=\frac{n\times C_{V}\times V_{V}\times 50.94}{1000}$$

(2) $$L=\frac{m_{1}-m_{2}}{m_{1}}\times 100\%$$

where m is the mass of vanadium in the solution, g; n is the volume of solution, mL; $C_{V}$ is the concentration of (NH_4_)_2_Fe(SO_4_)_2_ standard titration solution, mol/L; $V_{V}$ is the volume of (NH_4_)_2_Fe(SO_4_)_2_ standard titration solution consumed by 1 mL solution; 50.94 is the molar mass of vanadium, g/mol; L is the rate, %; $m_{1}$ is the mass of vanadium before precipitation or dissolution, g; $m_{2}$ the mass of vanadium after precipitation or dissolution, g.

### 2.3. Characterization

The concentration of vanadium in the liquid was determined through ammonium ferrous sulfate titration. The contents of manganese and vanadium in the solid samples were determined by inductively coupled plasma emission spectrometry (ICP-OES, Perkin Elmer Optima-4300 DV, Perkin Elmer Enterprise Management (Shanghai) Co., Ltd., Shanghai, China). The phases of the vanadium precipitation product obtained by manganese salt, vanadium precipitation products obtained by hydrolysis, and V_2_O_5_ were characterized using a powder X-ray diffraction system (XRD, X Pertpro, PANalytical B.V., Almelo, The Netherlands). The sodium content was quantified using an atomic absorption spectrophotometer (AAS, TAS-990, Beijing Purkinje general Instrument Co., Ltd., Beijing, China). The micro-morphology and distribution of the product were observed using a scanning electron microscope (Ultra Plus, Zeiss, Germany).

## 3. Experimental Results and Analysis

### 3.1. Vanadium Extraction by Manganese Salt

**Figure 2:**
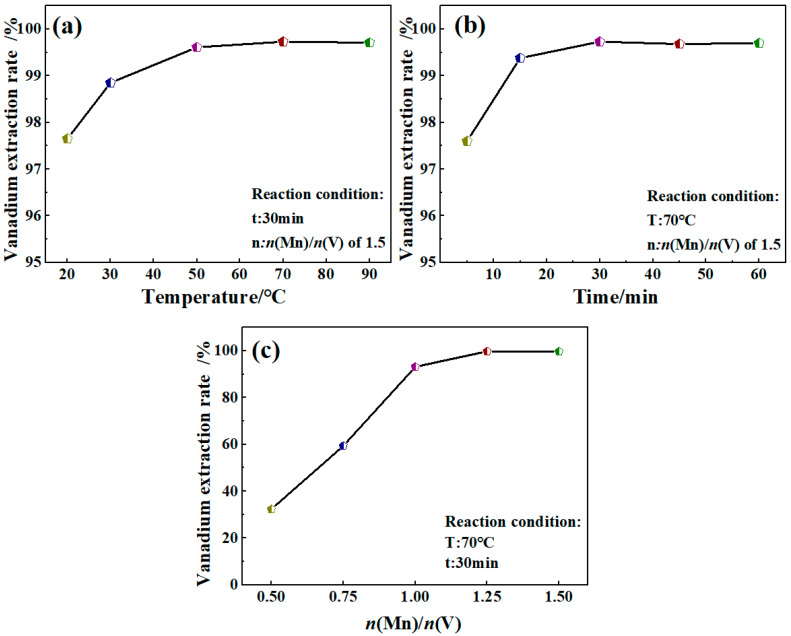
Effect of reaction conditions on rate of vanadium extraction by manganese salt. (**a**) Temperature; (**b**) Time; (**c**) *n*(Mn)/*n*(V).

Figure 2a depicts the impact of various reaction temperatures on the rate of vanadium extraction by manganese salt at the *n*(Mn)/*n*(V) ratio of 1.5 and a reaction time of 30 min. The results indicate that the rate of vanadium extraction by manganese salt steadily increased as the reaction temperature rose from 20 °C to 70 °C. This is because vanadium extraction by manganese salt is an endothermic reaction [37], meaning a rise in temperature will increase the molecular kinetic energy and the number of activated molecules, thus speeding up the reaction. The extraction rate of vanadium was 99.73% at 70 °C. The reaction temperature rose until it reached 90 °C, and the vanadium extraction rate remained basically unchanged. This result indicates that a reaction temperature of 70 °C is optimal for this process. Thus, vanadium extraction by manganese salt has a very high efficiency, proving the feasibility of its application in vanadium purification. Figure 2b illustrates the impact of varying reaction times on the vanadium extraction rate using manganese salt, maintaining the *n*(Mn)/*n*(V) ratio at 1.5 at a reaction temperature of 70 °C. The results indicate a gradual increase in the vanadium extraction rate from 97.60% to 99.73% as the reaction time extends from 5 to 30 min. This result further indicates that vanadium extraction from manganese salt has a very high efficiency. Extending the reaction time to 60 min does not further enhance the rate of vanadium extraction, which accelerates notably within the first 15 min and stabilizing at approximately 30 min. This stability behavior suggests that the optimal reaction duration is approximately 30 min. Figure 2c depicts how various *n*(Mn)/*n*(V) ratios affect the vanadium extraction rate when using manganese salt at a reaction temperature of 70 °C and at a duration of 30 min. The results represent the *n*(Mn)/*n*(V) ratio from 0.5 to 1.0; the vanadium extraction rate increases, indicating that the amount of manganese is insufficient and the vanadium in the solution cannot be completely extracted. As the *n*(Mn)/*n*(V) ratio increases from 1.0 to 1.25, the extraction rate also increases from 93.20% to 99.75%. However, when the ratio of *n*(Mn)/*n*(V) is 1.5, no further increase in speed is obse *n*(Mn)/*n*(V)rved. Therefore, the optimal ratio of *n*(Mn)/*n*(V) is 1.25.

**Figure 3:**
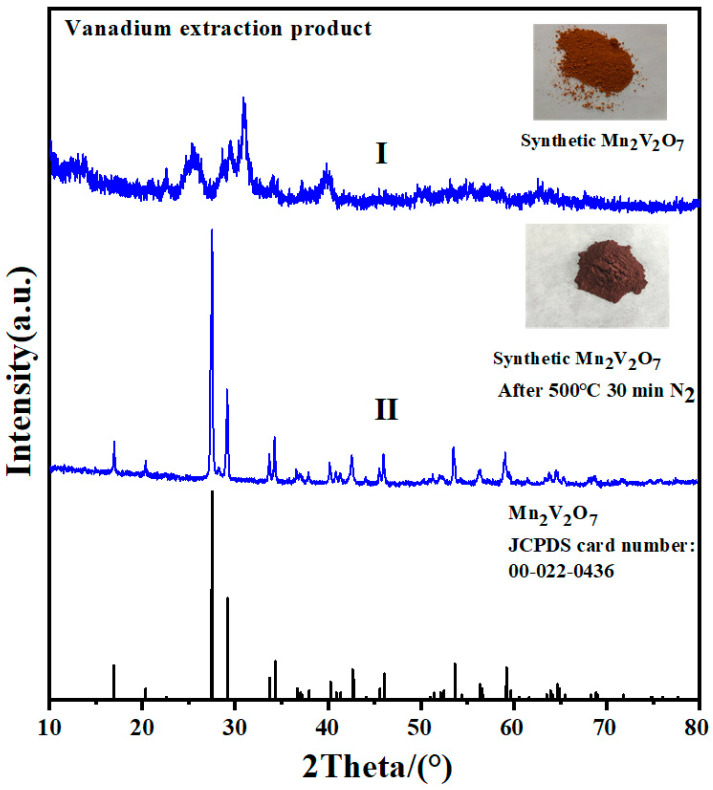
XRD characterization of vanadium extraction product by manganese salt.

The XRD diffraction peak of the vanadium extraction product obtained by manganese salt is depicted in Figure 3I, and the crystallinity of the product is poor. To improve the crystallinity and determine the phase composition of the product, the temperature was raised to 500 °C under an N_2_ atmosphere and maintained at that level for 30 min. This process helps to avoid other reactions involving the manganese vanadate products [37]. The XRD diffraction peak of the product after high-temperature crystallization is shown in Figure 3II. It closely aligns with the standard JCPDS card number 00-022-0436, confirming that the product is Mn_2_V_2_O_7_. No diffraction peaks of sodium-related compounds are observed in the figure: thus, vanadium remains in the solid phase in the form of Mn_2_V_2_O_7_, whereas sodium remains in the liquid phase in the form of Na_2_SO_4_, verifying the separation of vanadium and sodium. The reaction mechanism for vanadium extraction by manganese salt is expressed in Equation (3).

**Figure 4:**
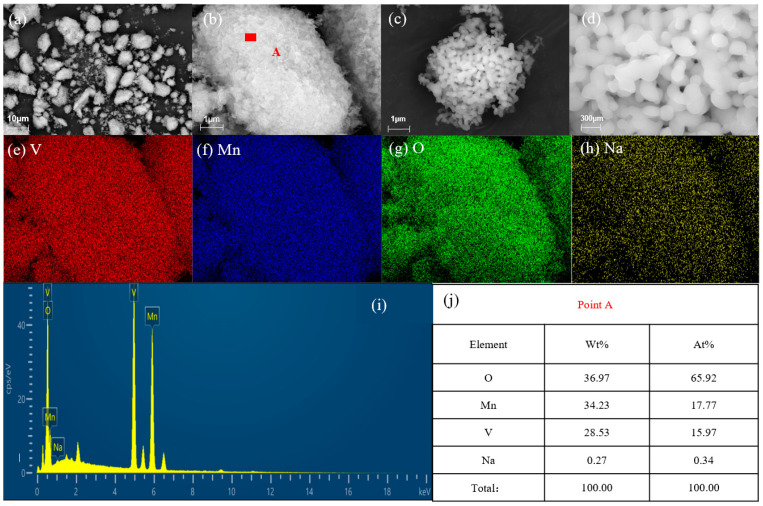
SEM and EDS characterization of vanadium extraction product by manganese salt. (**a**,**b**) Different magnification images of the product before crystallization; (**c**,**d**) Different magnification images of the product after crystallization; (**e**–**h**) elements mappings; (**i**,**j**) EDS analysis.

Figure 4a,b show SEM images of vanadium extraction products by manganese salts at different magnifications. This product has a loose, flaky microstructure. Figure 4c,d depict the vanadium extraction products by manganese salts after being enhanced by crystallization at 500 °C in an N_2_ atmosphere for 30 min. This process prevents other reactions between vanadium and manganese [38]. The vanadium precipitation product resulting from the manganese salt process has a compact, massive microstructure. A comparative analysis reveals that the product’s degree of crystallization is significantly improved. Figure 4e shows that vanadium is distributed in various regions of the product. Figure 4f shows that manganese is distributed in all regions of the product, which is consistent with the distribution region of vanadium. Figure 4g shows that the distribution regions of oxygen components and vanadium–manganese components coincide. Figure 4h shows that the sodium component area is dark, with obvious changes in brightness observed. This result indicates that sodium mainly adheres to the product because of incomplete washing during the suction filtration process. Figure 4i shows the X-ray energy spectrum of the product, which indicates three main energy spectrum peaks of vanadium, manganese, and oxygen; the energy spectrum peaks of sodium are few, and the peak intensity is low. Figure 4j is the EDS analysis of point A in Figure 4b, which shows that the mass fractions and atomic percentages of vanadium, oxygen, and manganese are similar to those of the three elements in Mn_2_V_2_O_7_; this observation is consistent with the XRD analysis results. Therefore, the product of vanadium precipitation by manganese salt is Mn_2_V_2_O_7_, confirming the separation of vanadium and sodium.

2MnSO_4_ + 2NaVO_3_ + 2NaOH = Mn_2_V_2_O_7_↓ + 2Na_2_SO_4_ + H_2_O (3)

Table 2 describes the chemical composition of the product. The product shows a mass ratio *m*(Mn)/*m*(V) of 1.055, which is equal to the mass ratio *m*(Mn)/*m*(V) of Mn_2_V_2_O_7_ of 1.058. The sodium content in the product is only 0.089%, indicating an efficient separation of vanadium and sodium. This extraction method effectively achieves the purification of vanadium in the NaVO_3_ solution and also lays a foundation for the subsequent vanadium precipitation by hydrolysis.

### 3.2. Acid Dissolution

**Figure 5:**
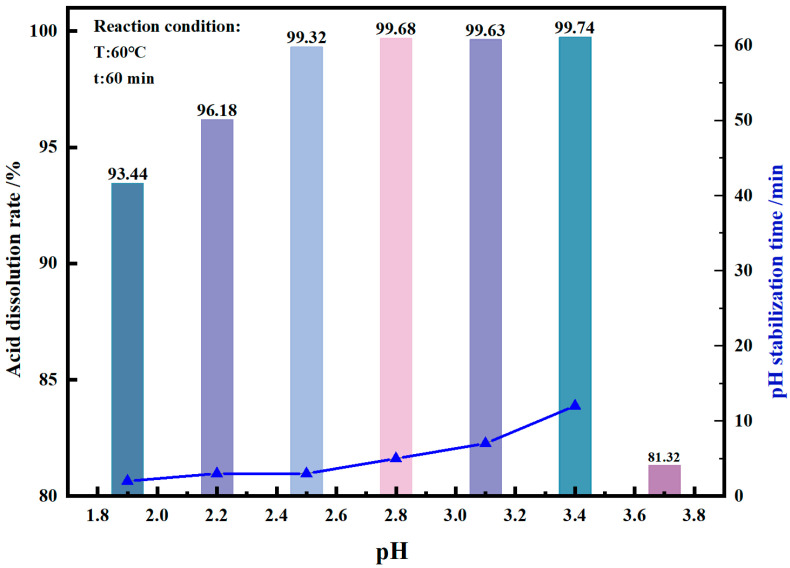
Effect of pH on acid dissolution rate of vanadium extraction product by manganese salt.

Figure 5 shows that the acid solubility of vanadium extraction products by manganese salt gradually decreases with a decrease in the pH value in the range of 1.9–2.5. This behavior occurs because as the pH decreases, the vanadium solution obtained through acid dissolution undergoes hydrolysis and precipitation, transforming vanadium from a liquid to a solid state. In the pH range of 2.8–3.4, the acid solubility of the vanadium extraction product by manganese salt is relatively stable above 99.60%. However, as the pH rises continuously, the time required for the pH of the solution to stabilize during acid dissolution (the time required from the start of the reaction to the slight changes in the pH of the solution) is prolonged. Meanwhile, the acid solubility of the vanadium extraction product is reduced at the same reaction time. For example, when the reaction time is 60 min, the acid solubility of the vanadium extraction product from manganese salt is only 81.32% at pH 3.7, which is because the leaching acidity decreases with the increase in pH, and the reaction rate decreases. Therefore, pH 2.8 is a suitable acid dissolution condition, considering that the vanadium solution will be hydrolyzed when the pH is too low. Moreover, the reaction rate will be reduced and the reaction time will be prolonged when the pH is too high. The acid dissolution mechanism of this product is expressed as Equation (4).

Mn_2_V_2_O_7_ + 6H^+^ = (VO_2_)_2_^2+^ + 2Mn^2+^ + 3H_2_O (4)

**Figure 6:**
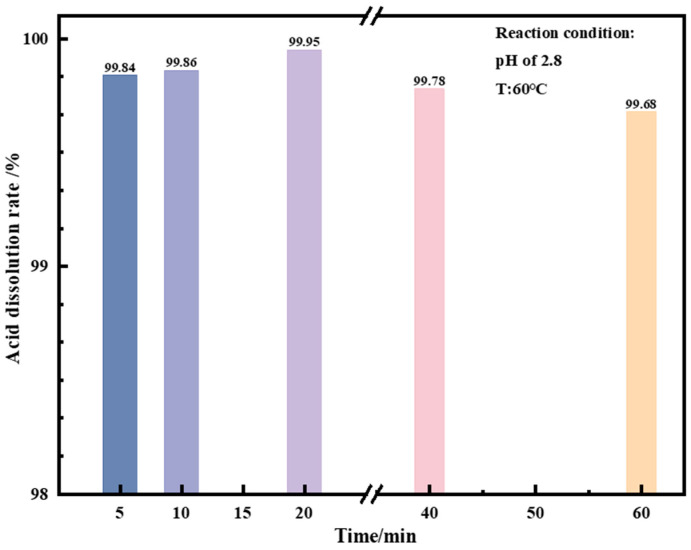
Effect of time on acid dissolution rate of vanadium extraction product by manganese salt.

Figure 6 illustrates the effect of reaction time on the acid solubility of the vanadium extraction product by manganese salt. This investigation was conducted at a reaction temperature of 60 °C and a reaction pH of 2.8. Within a reaction time of 5 min, 99.84% of the product was dissolved in the acid solution, and when the reaction time was extended to 20 min, 99.95% of the product was dissolved in the acid solution. As the reaction time further increased, the acid solubility decreased slightly, and the product solubility decreased to 99.78% in 40 min and 99.68% in 60 min. This is because as the reaction time is extended, weak hydrolysis and precipitation reactions occur in the vanadium solution, resulting in the transformation of liquid manganese vanadate solution after acid dissolution into solid hydrolysis and precipitation products, and the acid solubility decreases. To sum up, if the reaction pH is too low or the reaction time is too long, vanadium will undergo hydrolysis and precipitation, reducing the acid solubility of the vanadium extraction products from manganese salts. Therefore, the optimal dissolution conditions are a pH of 2.8 and a reaction time of 20 min, under which 99.95% of the product is dissolved in the acid solution.

### 3.3. Hydrolyzing and Precipitating Vanadium: Washing and Calcination

**Figure 7:**
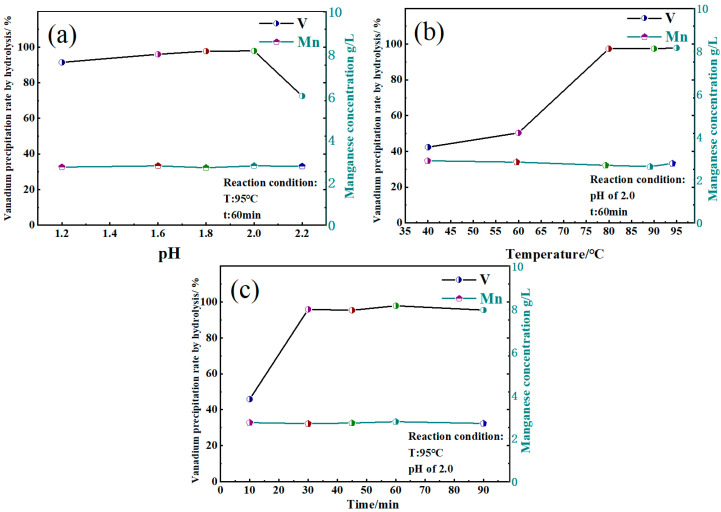
Effect of reaction conditions on vanadium precipitation rate by hydrolysis. (**a**) pH; (**b**) Temperature; (**c**) Time.

As depicted in Figure 7a, with the increase in pH, the rate of hydrolysis and precipitation of vanadium first increases and then decreases. At a pH of 2.0, the rate peaks at 97.87%. However, as the pH decreases to 1.6, the vanadium precipitation product begins to dissolve, decreasing the rate of vanadium precipitation by hydrolysis to 95.99%. When the pH is reduced to 1.2, a notable decrease is observed in the rate of vanadium precipitation by hydrolysis, reaching only 91.44%. This result shows that the vanadium precipitation product becomes soluble at high acidity. At a pH of 2.2, the hydrolysis precipitation rate of vanadium is only 72.51%, suggesting that a further increase in the pH becomes unfavorable for the hydrolysis precipitation of vanadium. It can be seen from Figure 7a that the separation of vanadium and manganese is relatively stable, and the change in pH has little effect on the concentration of manganese ions in the vanadium precipitation waste liquid. These manganese ions in the waste liquid can be recovered in the form of manganese sulfate by evaporation and crystallization. As shown in Figure 7b, when the reaction temperature rises from 40 °C to 80 °C, the precipitation rate of vanadium by hydrolysis increases significantly from 42.37% to 97.42%. From a reaction temperature of 80 °C to 95 °C, the hydrolysis and precipitation rate of vanadium increases from 97.42% to 97.87%, indicating that the hydrolysis and precipitation effect of vanadium is optimal in this temperature range. Further, with a rise in reaction temperature, the concentration of manganese ions in the waste liquid of vanadium precipitation increase slightly, indicating that more manganese ions could be recovered from the waste liquid. An evident increase is observed in the rate of vanadium precipitation by hydrolysis in Figure 7c, rising from 45.83% to 95.88% when the reaction time is extended from 10 min to 30 min. Extending the reaction time from 30 min to 60 min yields a relatively small increase in the rate of vanadium precipitation by hydrolysis, from 95.88% to 97.87%. However, as the reaction time is prolonged to 90 min, the rate of vanadium precipitation through hydrolysis decreases to 95.49%. This result shows that if the reaction time is too long, the precipitate obtained by hydrolysis will dissolve, decreasing the vanadium precipitation rate by hydrolysis. Meanwhile, Figure 7c shows that the reaction time has little effect on the concentration of manganese ions in the vanadium precipitation waste liquid. To sum up, the optimum conditions for vanadium precipitation by hydrolysis are a pH of 2.0, a reaction temperature of 95 °C, and a reaction time of 60 min.

**Figure 8:**
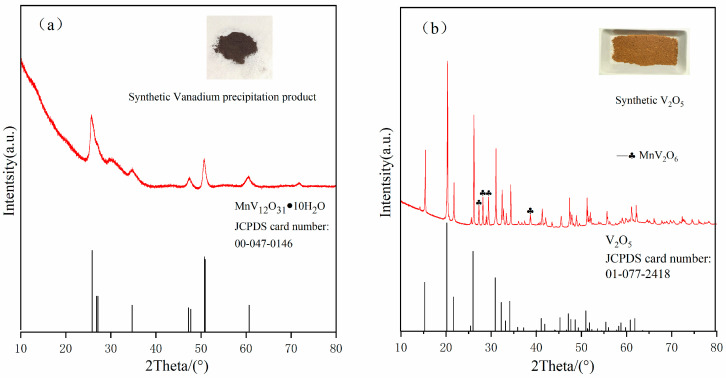
XRD images of the hydrolyzed product and calcined product. (**a**) Vanadium precipitation product; (**b**) V_2_O_5_.

**Figure 9:**
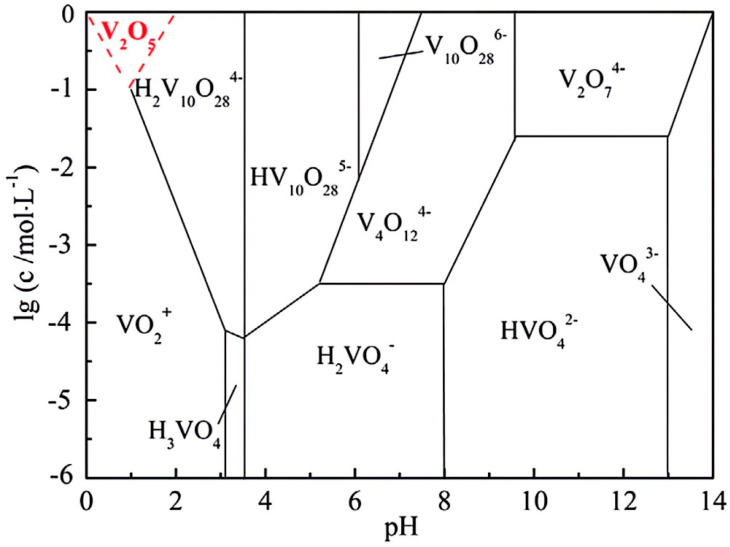
Existing form of vanadium at different pH values and concentrations [28].

Figure 8a shows the XRD pattern of the product of hydrolysis and vanadium precipitation under optimum conditions: a pH of 2.0, reaction temperature of 95 °C, and reaction time of 60 min. The diffraction peak is consistent with the standard peak of JCPDS card number 00-047-0146, indicating that the product of vanadium precipitation by hydrolysis is a compound of MnV_12_O_31_ 10H_2_O. As observed in Figure 9, at a pH of 2.0, the vanadium solution of 0.3394 mol/L (17.29 g/L) mainly exists in the form of H_2_V_10_O_28_^4−^, and the hydrolysis and vanadium precipitation reactions are expressed in Equation (5). However, at this pH, the H^+^ in the polymer is replaced by Mn^2+^, as illustrated in Equation (6). The results show that under these reaction conditions, a small amount of Mn^2+^ will replace H^+^ to participate in the hydrolysis and precipitation process of vanadium. Figure 8b shows the XRD pattern of the product obtained by calcining the hydrolyzed vanadium precipitation product at 550 °C for 2 h, and its diffraction peak is consistent with the standard JCPDS card number 01-077-2418. These results demonstrate that under these reaction conditions, a small amount of Mn^2+^ replaces H^+^ to participate in the hydrolysis and precipitation process of vanadium. However, there are also low-intensity diffraction peaks corresponding to MnV_2_O_6_. This is because manganese in the hydrolysis precipitation product, after calcination, exists in the form of MnV_2_O_6_.

6H_2_V_10_O_28_^4−^ + 24H^+^ = 5H_2_V_12_O_31_↓ + 13H_2_O (5)

H_2_V_12_O_31_ + Mn^2+^ = 5MnV_12_O_31_ + 2H^+^ (6)

**Figure 10:**
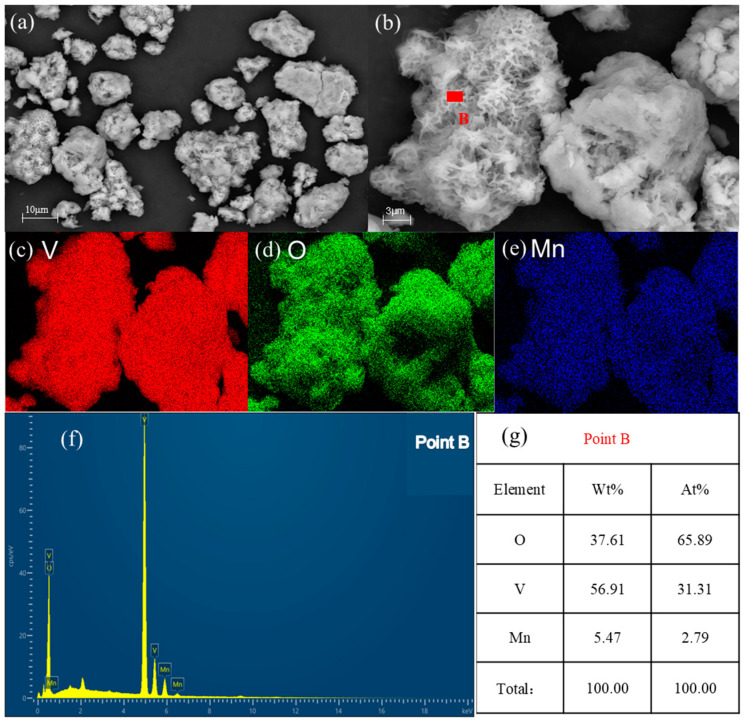
SEM and EDS images of a hydrolyzed product. (**a**,**b**) Different magnifications; (**c**–**e**) elements mappings; (**f**,**g**) EDS analysis.

Figure 10 shows SEM and EDS images of hydrolyzed vanadium precipitation products. Figure 10a,b are images of vanadium precipitation products at different magnifications, which reveal a network structure. Figure 10c,d show the surface scanning distribution of each element in the product. The product is mainly composed of vanadium, oxygen, and a small amount of manganese. Figure 10f shows the energy spectrum diagram of the product, demonstrating pronounced energy spectrum peaks for vanadium and oxygen, whereas those for manganese are comparatively weak. Figure 10g, on the other hand, shows the EDS results for point B in Figure 10b. The results indicate that the product is mainly composed of vanadium and oxygen, as well as a small amount of manganese, which is consistent with the XRD result.

**Figure 11:**
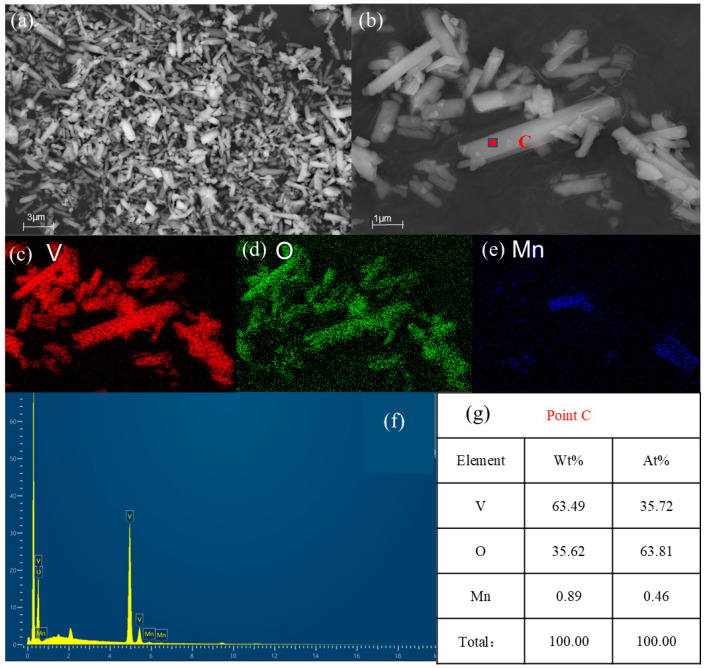
SEM and EDS images of a calcined product. (**a**,**b**) Different magnifications; (**c**–**e**) elements mappings; (**f**,**g**) EDS analysis.

Figure 11 presents the SEM and EDS diagrams of V_2_O_5_ obtained by calcining the hydrolysis vanadium precipitation product. Figure 11a,b V_2_O_5_ are images at different magnifications, which reveal a rod structure. Figure 11c,e show the surface scanning distribution of each element in the product. The images show that the product is primarily composed of vanadium and oxygen, including a small amount of manganese. Figure 11f shows the energy spectrum of the product, with obvious peaks of vanadium and oxygen, whereas the peaks of manganese are relatively weak. Figure 11g shows the EDS results of point C in Figure 11b. The product is mainly composed of vanadium and oxygen, including a small amount of manganese, which is consistent with the XRD results in Figure 8b.

Table 3 presents the chemical composition of V_2_O_5_ obtained by calcining hydrolysis and vanadium precipitation products. According to the previous analysis, manganese inevitably enters the hydrolysis solution during the vanadium precipitation by hydrolysis. Therefore, the V_2_O_5_ product contains 5.930% manganese and 0.048% sodium without washing with (NH_4_)_2_SO_4_ solution, and the purity of the V_2_O_5_ product is 92.21%. At the *n*(NH_4_^+^)/V_2_O_5_) ratio of 2, reaction temperature of 65 °C, reaction time of 5 min, and using the (NH_4_)_2_SO_4_ solution to wash twice to hydrolyze and precipitate vanadium, the V_2_O_5_ product contains 0.818% manganese and 0.006% sodium; its purity after manganese removal is 98.92%. NH_4_^+^ can replace Mn^2+^ and Na+ in the product of vanadium precipitation by hydrolysis. The pH of the (NH_4_)_2_SO_4_ solution after manganese removal is adjusted with ammonia water to precipitate Mn^2+^ as Mn(OH)_2_, and it is further adjusted with sulfuric acid for recycling. This process greatly reduces the production of ammonia nitrogen wastewater compared with the ammonium salt, direct vanadium precipitation process.

## 4. Conclusions

This study investigated the manganese salt pretreatment of NaVO_3_ solution, which yields vanadium extraction product by manganese salt and separates vanadium from sodium. This process establishes a foundation for the subsequent vanadium precipitation by hydrolysis from the vanadium solution. The manganese vanadate products undergo acid dissolution, facilitating the transformation of vanadium from solid to liquid, resulting in a manganese vanadate solution. The vanadium solution then undergoes precipitation by hydrolysis, followed by impurity removal and calcination to obtain the target product, V_2_O_5_. Through theoretical analysis and characterization, we draw the following conclusions:

(1) The optimum technological conditions for extracting vanadium from manganese salt of the NaVO_3_ solution are as follows: reaction temperature of 70 °C, reaction time of 30 min, and *n*(Mn)/*n*(V) ratio of 1.25, with a 99.75% vanadium extraction efficiency. The final product of vanadium extraction mediated by manganese salt is Mn_2_V_2_O_7_, which contains only 0.089% sodium, effectively completing the purification of vanadium.
(2) The most favorable conditions for the acid dissolution of Mn_2_V_2_O_7_ products are a pH of 2.8 and reaction time of 20 min, yielding a dissolution rate of 99.95%. Mn_2_V_2_O_7_ shows excellent solubility in a sulfuric acid medium.
(3) The optimum conditions for vanadium precipitation by hydrolysis are a pH of 2.0, reaction temperature of 95 °C, and reaction time of 60 min, yielding a vanadium precipitation rate of 97.87%. The purity of V_2_O_5_ after washing, manganese removal, and calcination reaches 98.92%. In addition, MnSO_4_ can be recovered by evaporation and crystallization. Meanwhile, the (NH_4_)_2_SO_4_ solution can also be recycled, greatly reducing the production of ammonia–nitrogen wastewater.

## Figures and Tables

**Table 1:** Experimental parameter design.

Parameter Setting	pH	T/°C	t/min	*n*(Mn)/*n*(V)
Vanadium extraction by manganese salt	11	20, 30, 50, 70, 90	5, 15, 30, 45, 60	0.5, 0.75, 1.0, 1.25, 1.5
Acid dissolution	1.9, 2.2, 2.5, 2.8, 3.1, 3.4, 3.7	60	5, 10, 20, 40, 60	—
Vanadium precipitation by hydrolysis	1.4, 1.6, 1.8, 2.0, 2.2	40, 60, 80, 90, 95	10, 30, 45, 60, 90	—

**Table 2:** Chemical composition of vanadium extraction product by manganese salt (wt%).

Element	V	Mn	Na
content	30.8	32.5	0.089

**Table 3:** Chemical composition of products before and after impurity removal (wt%).

Sample	V_2_O_5_	Mn	Na
V_2_O_5_ before manganese removal	92.21	5.930	0.048
V_2_O_5_ after manganese removal	98.92	0.818	0.006

## Data Availability

The original contributions presented in this study are included in the article. Further inquiries can be directed to the corresponding author.
